# Hypercalciuria in Postmenopausal Women With Reduced Bone Mineral Density Is Associated With Different Mineral Metabolic Profiles: Effects of Treatment With Thiazides and Anti-resorptives

**DOI:** 10.3389/fmed.2021.780087

**Published:** 2021-12-15

**Authors:** Federico Nicoli, Giorgia Dito, Gregorio Guabello, Matteo Longhi, Sabrina Corbetta

**Affiliations:** ^1^Department of Biotechnology and Translational Medicine, University of Milan, Milan, Italy; ^2^Endocrinology and Diabetology Service, IRCCS Istituto Ortopedico Galeazzi, Milan, Italy; ^3^Rheumatology Unit, IRCCS Istituto Ortopedico Galeazzi, Milan, Italy; ^4^Department of Biomedical, Surgical and Dental Sciences, University of Milan, Milan, Italy

**Keywords:** hypercalciuria, osteoporosis, PTH-parathyroid hormone, calcium, phosphate, hyperparathyroidism

## Abstract

Hypercalciuria may represent a challenge during the workup for osteoporosis management. The present study aimed: (1) to describe the phenotype associated with hypercalciuria in vitamin D-sufficient (serum 25 hydroxyvitamin D (25OHD) > 20 ng/ml) patients with osteopenia/osteoporosis; (2) to analyze the effects of thiazides and anti-resorptive drugs on urine calcium excretion (UCa), mineral metabolic markers, and bone mineral density. Seventy-seven postmenopausal women with hypercalciuria (Uca > 4.0 mg/kg body weight/24 h on two determinations) were retrospectively evaluated in a real-life setting. Median UCa was 5.39 (4.75–6.70) mg/kg/24 h. Kidney stones occurred in 32.9% of patients, who had median UCa similar to that of patients without kidney stones. Clustering analysis considering the three variables, such as serum calcium, phosphate, and parathormone (PTH), identified two main clusters of hypercalciuric patients. Cluster 1 (*n* = 13) included patients with a primary hyperparathyroidism-like profile, suggesting a certain degree of autonomous PTH secretion from parathyroid glands. Within cluster 2 (*n* = 61), two subgroups were recognized, cluster 2A (*n* = 18) that included patients with relatively increased PTH and normophosphatemia, and cluster 2B (*n* = 43) that included patients with the normal mineral profile. After a follow-up of 33.4 ± 19.6 months, 49 patients treated with thiazidic diuretics (TZD) were reevaluated; 20 patients were treated with hydrochlorothiazide (HCT; 12.5–37.5 mg/day), 29 with indapamide (IND; 1.50–3.75 mg/day). Any significant difference could be detected in all the parameters both basal and treated conditions between patients treated with HCT or IND. TZD induced a mean 39% reduction in UCa and 63.3% of patients obtained Uca < 4.0 mg/kg/24 h, independent of their mineral metabolic profile. Moreover, TZD induced a significant decrease in PTH levels. TZD-treated patients normalizing UCa experienced an increase in bone mineral densities when concomitantly treated with anti-resorptives, while any gain could be observed in TZD-treated patients with persistent hypercalciuria. Finally, multiple regression analysis showed that UCa reduction was at least in part related to denosumab treatment. In conclusion, in postmenopausal osteoporotic women, hypercalciuria is associated with kidney stones in about one-third of patients and with a wide range of impaired PTH secretion, determining a diagnostic challenge. TZD efficiently reduces UCa and normalization contributes to increasing anti-resorptives positive effect on bone mineral density.

## Introduction

The role of hypercalciuria in kidney stones development has been extensively investigated in the general population (1) and patients with endocrine disorders, mainly in patients with primary hyperparathyroidism (2), while poor data are available about its role in bone metabolism derangement in the set of postmenopausal and aging osteoporosis. Hypercalciuria is frequently diagnosed during the workup for osteoporosis management (3), where it can be detected in up to one-third of patients with osteoporosis. Several metabolic disorders and medical treatments may promote hypercalciuria, such as primary hyperparathyroidism (PHPT), hyperthyroidism, Paget's disease, myeloma, malignancy, immobility, accelerated osteoporosis, sarcoidosis, and renal tubular acidosis. Besides these conditions, hypercalciuria often recognizes a genetic predisposition manifesting as kidney stones in young patients and with osteoporosis and fragility fractures in postmenopausal women and elder men. Idiopathic hypercalciuria is determined by polymorphic variants/mutations of the calcium sensing receptor (*CASR*) gene (4–6), mutations of *SLC34A1*, and *SLC34A4* genes (7, 8), polymorphic variants of claudins (9), genes involved in calcium reabsorption at the tubular level.

The syndrome of idiopathic hypercalciuria has been described as including low normal serum phosphate, normocalcemia, hypercalciuria, and kidney stones (10). Those individuals with fasting hypercalciuria along with an increased or high/normal parathormone (PTH) are defined as having “renal leak” as the primary mechanism, with the basic abnormality resting within the nephron (10). In clinical practice, distinguishing among intestinal hyperabsorptive hypercalciuria, renal leak hypercalciuria, and idiopathic or fasting hypercalciuria can be difficult (10). Moreover, increased renal calcium excretion is a well-known cause of secondary hyperparathyroidism, where PTH induced bone demineralization by increasing bone resorption. Hypercalciuria is associated with PHPT (11); nonetheless, in a subset of patients with PHPT, surgical resolution of hyperparathyroidism does not associate with urine calcium excretion (UCa) normalization (12). Therefore, it emerges as a spectrum of PTH-related disorders associated with hypercalciuria and with serum calcium levels ranging from normocalcemia to hypercalcemia. It should be also considered that vitamin D and kidney function are important determinants of circulating PTH levels (13). Taken together, all these factors related to hypercalciuria may make it difficult to distinguish PHPT, which may benefit from parathyroidectomy, from non-autonomous PTH-related conditions, where surgery should be avoided as highly probable ineffective in resolving hypercalciuria. In the set of patients with osteoporosis, this diagnostic challenge represents a clinically unmet need, that often increases the number of diagnostic procedures, biochemical tests, and specialist consults proposed to the patients.

Besides the diagnostic challenge, the management and treatment of hypercalciuria are not well defined. Thiazidic diuretics (TZD) are widely used for the management of hypercalciuria among stone-forming patients. TZD stimulates proximal tubular sodium reabsorption as a compensation of their natriuretic action in the distal tubule, resulting in enhanced proximal passive calcium transport (14). Although the effects of different TZD should be relatively similar in terms of prevention of stone recurrence, their potency and side effects may differ. Data concerning the metabolic and bone effects of these agents are limited among recurrent nephrolithiasis patients with hypercalciuria (15). Conceptually, TZD increases tubular calcium reabsorption, thus providing protection against hypercalciuria, and with that may raise serum calcium, suppress PTH secretion, and improve bone metabolism. The additional hypocalciuric effect may be observed with the use of potassium-sparing diuretics (16).

Finally, it should be considered that increased calcium excretion may be due to the increased bone reabsorption and benefit by treatment with anti-resorptive drugs, such as bisphosphonates and denosumab.

In patients affected with osteopenia/osteoporosis, the role of hypercalciuria needs to be elucidated and its management with TZD and/or anti-resorptive drugs needs to be defined.

Focusing on postmenopausal women with reduced bone density, the present study aimed: (1) to describe the different phenotypes associated with hypercalciuria in patients with osteopenia/osteoporosis, pointing on kidney, bone, and biochemical features; and (2) to analyze the efficacy of TZD treatment on reduction of renal calcium excretion, normalization of circulating PTH levels, hypercalcemia development, and on bone mineral density.

## Materials and Methods

### Data

Clinical data derived from Osteoregistry, a database collecting real life data from patients referred for osteoporosis management to the third level Endocrinology Service and Rheumatology Unit at IRCCS Istituto Ortopedico Galeazzi in Milan, Italy. All patients gave their informed consent. The study was approved by the local ethical committee. All study procedures were performed in compliance with the laws and regulations governing the use of human subjects (Declaration of Helsinki).

### Study Design

This is a retrospective study enrolling 77 osteopenic/osteoporotic women affected with persistent hypercalciuria. Hypercalciuria was defined as UCa > 4.0 mg/kg body weight/24 h on at least two determinations on unrestricted diet. Diagnosis of hypercalciuria was established during clinical, biochemical, and hormonal workup for the evaluation of osteoporosis. Exclusion criteria were: male gender; premenopausal women; diagnosis of overt or subclinical hyperthyroidism, endogenous hypercortisolism, Paget's disease, multiple myeloma, malignancy, immobility, sarcoidosis, renal tubular acidosis, corticosteroids, loop diuretics, and hypoalbuminemia.

All patients were investigated by collecting history and anthropometric parameters (weight, height, and body mass index [BMI]). Biochemical profile, such as serum total calcium, phosphate, PTH, creatinine, 25 hydroxyvitamin D (25OHD), total alkaline phosphatase (ALP), and carboxy-terminal collagen crosslinks (βCTX). In patients treated with denosumab, the plasma PTH was assessed at 5 months from the last administration. Plasma ionized calcium was determined in patients with hypercalcemia. The estimated glomerular filtration rate was calculated for each patient according to the Chronic Kidney Disease-Epidemiology Collaboration (CKD-EPI) formula (17). Renal calcium excretions were measured on 24 h urine collection on at least two distinct occasions in all patients under unrestricted diet. Serum 25OHD levels were measured in all patients at every clinical reassessment and corrected by supplementation with cholecalciferol or calcifediol to maintain serum 25OHD levels above the threshold of 20 ng/ml. Anti-transglutaminase autoantibodies were assessed to exclude celiac disease when signs of malabsorption were detected, namely, anemia due to iron insufficiency, or in the presence of other autoimmune manifestations, namely, atrophic gastritis, vitiligo, rheumatoid arthritis, or autoimmune thyroid diseases.

Kidney stones were detected by kidney ultrasound imaging or as a history of symptomatic kidney stones. All patients underwent dual energy X-ray densitometry (DXA) evaluation for the measurement of bone mineral density at lumbar and femur sites (femur neck and hip) by Hologic or Lunar devices. DXA examination was repeated after not less than 18 months. All patients were evaluated for the occurrence of morphometric vertebral fractures by routine dorsal and lumbar X-ray imaging. In addition, clinical fragility fractures were recorded.

### Statistical Analysis

Unsupervised hierarchical clustering analysis by Euclidean wardd2 was performed to identify specific profiles based on mineral metabolic parameters. Principal component analysis (PCA) was calculated to confirm the clustering.

Non-parametric Kruskall–Wallis one-way ANOVA adjusted for multiple testing was performed to analyze the differences among the three mineral metabolic profile-based clusters when variables failed the normality test. Normally distributed variables were analyzed by ordinary one-way ANOVA.

Differences in the biochemical parameters between the two groups were detected by using paired *t*-test or Mann–Withney test for parametric and non-parametric parameters, respectively. Differences in percentages between the two groups were tested by Fisher's exact test. Linear multiple regression considering the treatment with bisphosphonates or denosumab concomitant with TZD as independent variables, and the changes in UCa as the dependent variable was used to test the hypothesis that anti-resorptive drugs may contribute to reducing UCa. Parametric data were presented as mean ± SD, while non-parametric variables were presented as median (range interquartile). Data were analyzed by using Past 3.14 (18) and Prism 6.0. A *P*-value of < 0.05 was considered statistically significant.

## Results

### Clinical and Biochemical Features of Patients With Reduced Bone Mineral Density and Persistent Hypercalciuria

Clinical and biochemical features of the enrolled postmenopausal women are presented in Table 1. About half of patients were supplemented with cholecalciferol, while 17% of patients were treated with bisphosphonates. All patients had circulating 25OHD levels above 20 ng/ml at the time of basal evaluation.

**Table 1:** Clinical and biochemical features of postmenopausal women with low bone mineral density and persistent hypercalciuria.

**Parameters**	**Normal values**	
*N*		77
Age (years)		63.0 (57.0–69.0)
BMI (kg/m^2^)		23.5 (21.9–27.4)
HRT (%)		7 (9.2)
Active smoke (%)		14 (18.0)
Arterial blood hypertension (%)		7 (9.2)
Familiarity for fragility fractures (%)		15 (19.5)
Cholecalciferol (%)		35 (45.5)
Bisphosphonates (%)		13 (16.9)
***Circulating mineral and bone markers***		
Serum Ca (mg/dl)	8.4–10.4	9.6 (9.2–10.0)
Serum P (mg/dl)	3.0–5.0	3.3 (3.0–3.7)
Plasma PTH (pg/ml)	15.0–65.0	35.0 (42.0–82.1)
Serum 25OHD (ng/dl)		35.0 (27.1–41.0)
Serum total ALP (U/L)	40.0–120.0	67.0 (55.8–92.3)
Serum βCTX (ng/ml)		0.510 (0.208–0.767)
***Kidney features***		
Serum Cr (mg/dl)	<0.80	0.70 (0.60–0.78)
eGFR (ml/min)	>60.0	93.2 (87.9–98.1)
UCa (mg/24h)	<400.0	321.0 (277.0–405.6)
UCa (mg/kg/24h)	<4.0	5.39 (4.75–6.70)
Urine P (mg/kg/24h)		815.0 (585.0–995.5)
Kidney stones (%)		25 (32.9)
***Bone features***		
L1-L4 T-score	>-2.5 SD	−2.80 (−3.58, −2.03)
Femur neck T-score	>-2.5 SD	−2.20 (−2.80, −1.80)
Total hip T-score	>-2.5 SD	−1.87 (−2.50, −1.35)
Osteoporosis at L1-L4 level (%)	<-2.5 SD	46 (60.0%)
Osteopenia at L1-L4 level (%)	<-1.0, >-2.5 SD	27 (35.0%)
Osteoporosis at femur neck level (%)	<-2.5 SD	28 (36.4%)
Osteopenia at femur neck level (%)	<-1.0, >-2.5 SD	45 (58.4%)
Fractures (%)		37 (48.7)

*n, number; BMI, body mass index; HRT, hormone replacement therapy; Ca, total calcium; P, phosphate; PTH, parathormone; 25OHD, 25 hydroxyvitamin D; ALP, alkaline phosphatase; βCTX, β carboxy-terminal collagen crosslinks; Cr, creatinine; eGFR, estimated glomerular filtration rate calculated according to the KDIGO CKD-EPI formula (17); UCa, urine calcium excretion. Parameters in the whole series were not normally distributed; they are presented as median (range interquartile) or percentages*.

Kidney features Median (interquartile range) 24 h-UCa was 5.39 (4.75–6.70) mg/kg/24 h. Kidney stones were detected anamnestically or by imaging in about one-third of patients, whose UCa was similar to that of patients without kidney stones [5.33 (4.67, 7.04) vs. 5.40 (4.77, 6.68) mg/kg/24 h; *P* = 0.814]. Indeed, patients with kidney stones had lower serum total calcium [9.2 (9.0, 9.8) vs. 9.7 (9.3, 10.0) mg/dl, *P* = 0.035], while patients with hypercalciuria without kidney stones had significantly lower median lumbar T-score than that detected in patients with kidney stones [−2.35 (−3.1, −1.85) vs. −3.1 (−3.7, −2.3); *P* = 0.018].

Bone features DXA scan diagnosed osteoporosis (T-score <-2.5 SD) in 60.0% and osteopenia (−1.0 < T-score <-2.5 SD) in 35.0% of patients at lumbar site and osteoporosis in 36.4% and osteopenia in 58.4% of patients at femur neck. At least one fragility fracture occurred in 48.7% of patients [median 2.0 (1.0, 3.0)]. UCa did not differ between fractured and unfractured patients [5.40 (4.75, 6.89) vs. 5.34 (4.74, 6.43) mg/kg/24 h; *P* = 0.531], though fractured patients were older (65.6 ± 8.3 vs. 61.0 ± 6.3 years; *P* = 0.007), heavier [25.2 (22.4, 29.5) vs. 22.9 (21.6, 24.8) kg/m^2^; *P* = 0.020], and with more severe reduced bone densities [L1–L4 T-score −3.15 (−3.70, −2.50) vs. −2.50 (−3.30, −1.93), *P* = 0.036; femur neck T-score −2.40 (−2.98, −1.90) vs. −2.10 (−2.70, −1.70), *P* = 0.013; femur hip T-score −2.20 (−2.80, −1.50) vs. −1.50 (−2.20, −1.10), *P* = 0.003].

### Mineral Metabolic Parameters-Related Clustering of Hypercalciuric Women With Reduced Bone Mineral Density

**Figure 1:**
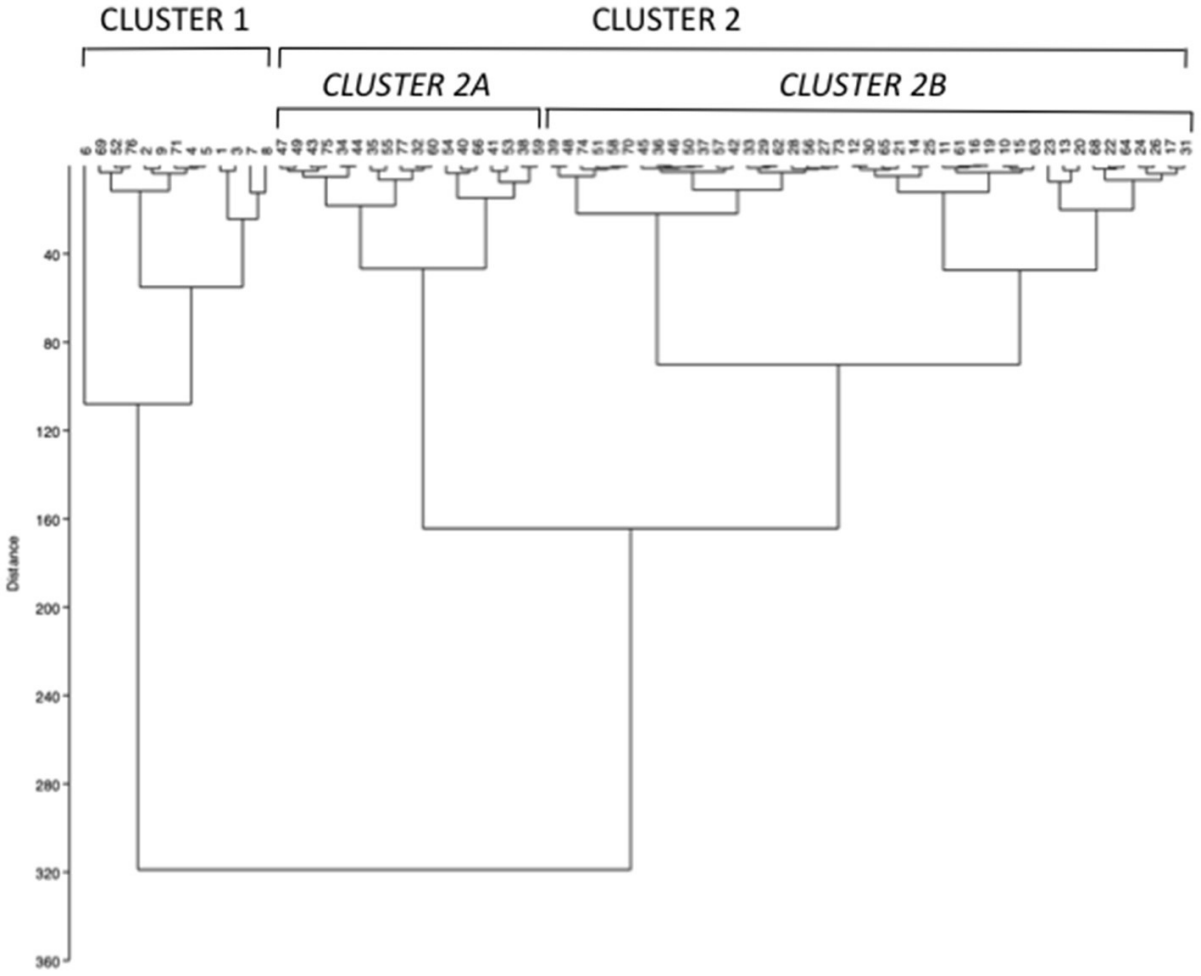
Unsupervised hierarchical cluster analysis by Euclidean wardd2 identifying two main clusters (cluster 1 and 2) based on the mineral metabolic parameters serum calcium, phosphate, and parathormone (PTH) at basal evaluation. A second order of clusterization identified two subgroups in cluster 2, named cluster 2A and 2B.

The unsupervised hierarchical cluster analysis by Euclidean wardd2 considering the three correlated variables serum calcium, phosphate, and PTH, identified two main clusters of patients with hypercalciuria (Figure 1): cluster 1 included 13 patients, cluster 2 included 61 patients, while three patients were excluded from the analysis due to unavailability of serum phosphate determinations. The second order of clusterization identified, in the set of cluster 2, two subgroups, indicated as cluster 2A and cluster 2B. The main PCA component explaining about 99% of the variance was determined by PTH values. All patients in cluster 1 presented the PTH levels higher than the upper limits of the normal range, while all patients in cluster 2B presented the PTH levels within the normal range. Patients in cluster 1 had significantly higher serum total calcium, lower phosphate, and higher PTH levels than patients in cluster 2B (Table 2), suggesting a condition of primary hyperparathyroidism due to inappropriate PTH release. Indeed, among patients in cluster 1, three women (23%) had serum total calcium levels above the upper limit of the normal range (10.4 mg/dl), of which only one had ionized calcium at the upper limit of the normal range (1.30 mmol/L; nv. 1.18–1.30 mmol/L). Among patients in cluster 2, two women in cluster 2A (11.1%) and two women in cluster 2B (4.7%) presented serum calcium levels above the upper normal limit, of which one in cluster 2A had concomitantly increased ionized calcium (1.36 mmol/L).

**Table 2:** Clinical and biochemical features of the clusters of postmenopausal women with reduced bone mineral density and persistent hypercalciuria identified on the base of serum calcium, phosphate and PTH.

**Parameters**	**N.V**.	**Cluster 1**	**Cluster 2**		**P**
			**Cluster 2A**	**Cluster 2B**	
*n*		13	18	43	
Age (years)		63.5 ± 7.5	64.0 ± 7.5	62.7 ± 7.9	0.820
BMI (kg/m^2^)		24.8 (23.4, 29.9)	23.5 (21.7, 29.1)	22.9 (21.8, 26.7)	0.157
Cholecalciferol (%)		9 (69.2)	14 (77.8)	31 (72.1)	0.689
Bisphosphonates (%)		3 (23.0)	7 (38.9)	11 (25.6)	0.452
Denosumab (%)		1 (7.7)	1 (5.5)	1 (2.3)	0.414
Serum Ca (mg/dl)	8.4–10.4	10.0 (9.4, 10.8)	9.5 (9.0, 10.3)	9.5 (9.2, 9.8)*	**0.049**
Serum P (mg/dl)	3.5–5.0	2.8 (2.5, 3.2)	3.3 (2.8, 3.6)	3.5 (3.2, 3.8)**	**0.016**
Plasma PTH (pg/ml)	15.0–65.0	121.0 (116.0, 142.3)	80.5 (72.4, 91.6)	47.0 (37.7, 58.0)°§	**<0.001**
Serum 25OHD (ng/dl)	>20	36.4 (28.2, 44.1)	31.4 (26.4, 40.0)	36.1 (27.0, 40.0)	0.437
Serum ALP (U/L)	40–120	84.9 ± 39.4	65.8 ± 7.6	70.5 ± 22.5	0.188
Serum CTX (ng/ml)		0.805 (0.198, 0.837)	0.510 (0.066, 0.620)	0.463 (0.235, 0.711)	0.415
Serum Cr (mg/dl)	<0.9	0.75 (0.72, 0.77)	0.68 (0.60, 0.81)	0.68 (0.59, 0.79)	0.449
eGFR (ml/min)	>60	94.1 (93.2, 95.6)	91.1 (90.3, 98.5)	92.8 (86.3, 98.9)	0.912
UCa (mg/24h)	<400	409.2 (335.0, 487.0)	361.0 (274.8, 431.2)	303.0 (269.0, 360.0)#	**0.002**
UCa (mg/kg/24h)	<4.0	5.64 (5.30, 8.19)	5.8 (5.04, 6.67)	5.10 (4.60, 6.69)	0.080
Kidney stones (%)		4 (30.8)	4 (22.2)	15 (34.9)	0.381
L1-L4 T-score		−2.80 (−3.75, −2.25)	−3.30 (−3.63, −2.75)	−2.65 (−3.33, −1.90)	0.394
Femur neck T-score		−2.00 (−2.45, −1.65)	−2.40 (−2.95, −1.90)	−2.30 (−2.80, −1.79)	0.316
Femur hip T-score		−1.65 (−2.28, −1.25)	−2.10 (−2.80, −1.40)	−1.80 (−2.40, −1.15)	0.482
Fractures (%)		5 (38.5)	10 (55.5)	22 (51.2)	0.473

*N.V., normal values; n, number; Ca, total calcium; P, phosphate; PTH, parathormone; 25OHD, 25hydroxyvitamin D; ALP, alkaline phosphatase; CTX, carboxy-terminal collagen crosslinks; Cr, creatinine; eGFR, estimated glomerular filtration rate calculated according the KDIGO CKD-EPI formula (17); UCa, urine calcium excretion. Normally distributed variables are presented as mean ± standard deviation and analyzed by ordinary One-way ANOVA; non normally distributed variables are presented as median (range interquartile) and analyzed by One-way ANOVA Kruskal-Wallis test; P-value with statistical significance are indicated in bold*.

* *P = 0.048 vs. cluster 1 by Dunn's multiple comparison test*.

** *P = 0.001 vs. cluster 1 by Dunn's multiple comparison test*.

° *P < 0.0001 vs. cluster 1 by Dunn's multiple comparison test*.

§ *P < 0.0001 vs. cluster 2A by Dunn's multiple comparison test*.

# *P = 0.0015 vs. cluster 1 by Dunn's multiple comparison test*.

Moreover, cluster 1 patients showed a tendency to present higher median renal calcium excretion than that in patients included in cluster 2B, though it did not reach the statistical significance. The prevalence of kidney stones was similar in the three clusters. Similarly, lumbar and femur bone mineral densities, as well as fractures prevalence, did not differ among clusters.

Age, BMI, kidney function, circulating bone markers, vitamin D status (evaluated as serum 25OHD levels), cholecalciferol supplementation, and prevalence of ongoing treatment with bisphosphonates or denosumab at time of basal evaluation, were similar among the three clusters (Table 2).

Therefore, cluster analysis suggested a continuum of PTH-dependent alterations in mineral metabolic parameters associated with hypercalciuria in postmenopausal women with reduced bone mineral density.

### Effects of Treatment With TZD

Forty-nine patients were treated with TZD and were re-evaluated after 33.4 ± 19.6 months. Twenty patients were treated with hydrochlorothiazide (HCT) at a dose ranging 12.5–37.5 mg/day, while 29 patients were treated with indapamide at a dose ranging 1.50–3.75 mg/day. Any significant difference could be detected between patients treated with HCT and indapamide in the analyzed parameters at both basal and on treatment evaluations. In particular, patients treated with HCT or indapamide showed similar reductions in renal calcium excretion (Table 3). Therefore, patients treated with HCT or indapamide were analyzed as a unique series. At the time of enrollment, 71.4% of patients were supplemented with cholecalciferol, while about a quarter was treated with bisphosphonates for a period not longer than 12 months. At the time of re-evaluation on TZD treatment, almost all patients were supplemented with cholecalciferol, all patients treated with bisphosphonates maintained their therapy, while about one-third started treatment with denosumab.

**Table 3:** Comparison of the effects of indapamide (IND) vs. hydrochlorothiazide (HCT) in postmenopausal women with reduced BMD and persistent hypercalciuria.

	**BASAL**			**TREATED**		
**Parameters**	**IND**	**HCT**	**P**	**IND**	**HCT**	***P***
*n*	29	20		29	20	
Follow up (months)	30.0 (20.5, 38.0)	36.0 (19.0, 48.0)	0.259			
BMI (kg/m^2^)	25.0 ± 4.6	26.7 ± 5.2	0.231			
Age (years)	61.8 ± 7.4	62.6 ± 6.4	0.682			
Serum Ca (mg/dl)	9.6 ± 0.6	9.7 ± 0.6	0.513	9.7 (9.3, 10.0)	9.6 (9.3, 10.1)	0.820
Serum P (mg/dl)	3.3 (3.1, 3.6)	3.4 (2.8, 3.8)	0.896	3.2 (2.8, 3.4)	3.5 (3.1, 3.7)	0.087
Plasma PTH (pg/ml)	62.0 (54.0, 88.4)	53.1 (43.3, 93.7)	0.371	54.4 (48.2, 72.1)	48.5 (36.0, 80.0)	0.548
25OHD (ng/dl)	37.0 ± 12.3	35.7 ± 15.2	0.737	41.8 (33.0, 47.7)	42.3 (35.0, 63.0)	0.569
UCa (mg/kg/24 h)	6.0 ± 1.7	5.6 ± 1.1	0.355	3.8 ± 1.4	3.2 ± 1.1	0.139
Δ UCa (mg/kg/24 h)				−0.35 (−0.49, −0.26)	−0.45 (−0.52, −0.27)	0.272
UCa <4 mg/kg/24 h (%)				19 (65.5)	14 (70)	0.768
eGFR (ml/min/1.73 m^2^)	93.8 (90.8, 98.4)	92.5 (88.8, 98.9)	0.865	90.7 (86.1, 96.8)	91.7 (87.6, 96.2)	0.838
L1-L4 T-score	−2.83 ± 1.05	−2.76 ± 1.13	0.803	−2.68 ± 1.00	−2.33 ± 1.15	0.327
Femur neck T-score	−2.28 ± 0.66	−2.26 ± 0.65	0.919	−2.27 ± 0.81	−2.08 ± 0.75	0.448
Femur hip T-score	−1.90 ± 0.78	−1.88 ± 0.96	0.940	−1.85 ± 0.73	−1.73 ± 0.73	0.606

*Basal, patients evaluated at the enrolment; treated, patients evaluated on treatment; IND, indapamide; HCT, hydrochlorothiazide; n, number; BMI, body mass index; Ca, total calcium; P, phosphate; PTH, parathormone; 25OHD, 25hydroxyvitamin D; UCa, urine calcium excretion; Δ UCa, difference between treated and basal UCa, normalized for basal UCa; eGFR, estimated glomerular filtration rate calculated according the KDIGO CKD-EPI formula (17). Normally distributed variables are presented as mean±standard deviation and analyzed by T-test of Student; non normally distributed variables are presented as median (range interquartile) and analyzed by Mann-Whitney test*.

#### Effects of TZD on UCa and PTH Levels

**Figure 2:**
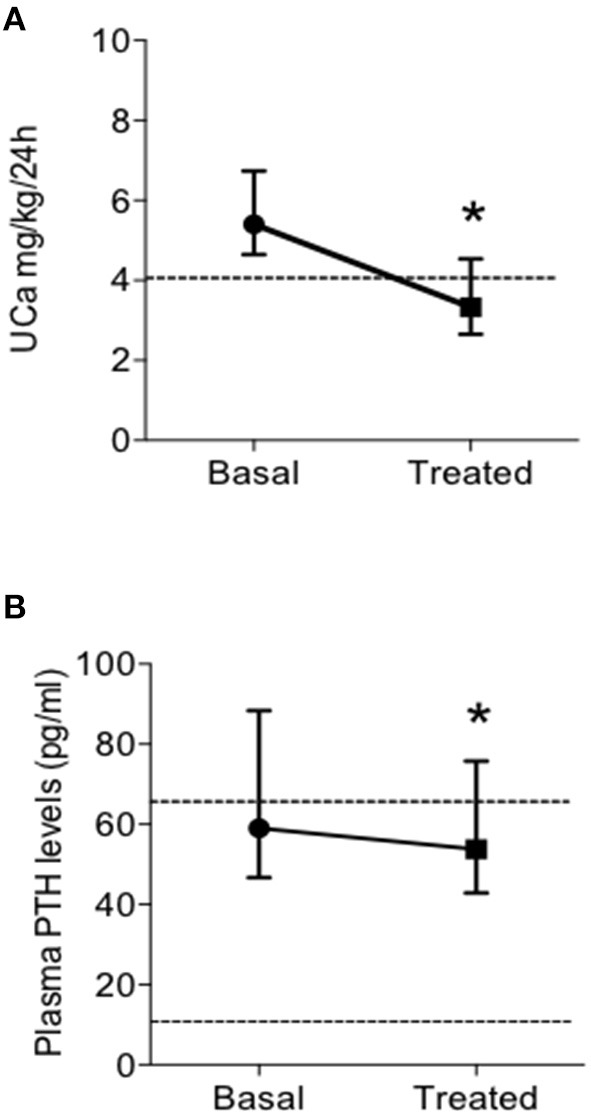
Effects of treatment with thiazidic diuretics (TZD) on urine calcium excretion (UCa) **(A)** and plasma PTH levels **(B)** in postmenopausal women with reduced bone mineral density and persistent hypercalciuria (*n* = 49). UCa, urine calcium excretion; and PTH, parathormone. ^*^, *P* = 0.005.

Patients on TZD treatment showed a significant reduction of renal calcium excretion with a mean decrease of 39% of basal levels (Figure 2A), determining normalization of the urine calcium levels in 31 (63.3%) patients. Moreover, TZD treatment reduced median circulating PTH levels (Figure 2B). Considering the concomitant treatment with bisphosphonates or denosumab as independent variables, linear multiple regression showed that the TZD-induced changes in UCa is significantly related to treatment with denosumab (coefficient −0.147, *P* = 0.016).

The same pattern of response was observed considering the subset of patients treated with TZD in absence of concomitant bone anti-resorptive therapy (both bisphosphonates and denosumab; *n* = 19) (Table 4). In particular, TZD treatment determined significant reductions of UCa and PTH levels. At variance, a mild increase (0.3 mg/dl) of serum total calcium could be detected. Of note, the entity of the TZD-induced PTH reduction was significantly higher in patients not concomitantly treated with anti-resorptive agents (bisphosphonates, denosumab, Δ basal PTH-post TZD PTH levels, 20.3 ± 24.6 vs. 6.2 ± 22.4 pg/ml, *P* = 0.045 by *T*-Student's test).

**Table 4:** Effects of thiazidic diuretics (TZD) treatment on biochemical parameters in postmenopausal women with reduced BMD and persistent hypercalciuria not treated with anti-resorptives.

**Parameters**	**BASAL**	**TZD**	**Δ Basal-Treated**	***P***
N	19	19		
Serum Ca (mg/dl)	9.5 ± 0.6	9.8 ± 0.5	−0.3	**0.032**
Serum P (mg/dl)	3.29 ± 0.52	3.29 ± 0.52	0.01	0.967
Plasma PTH (pg/ml)	78.3 ± 38.9	57.9 ± 25.8	20.3	**0.002**
25OHD (ng/dl)	37.7 ± 16.2	42.7 ± 9.4	−4.94	0.290
UCa (mg/kg/24h)	5.84 ± 1.62	3.90 ± 1.14	1.94	**<0.001**
eGFR (ml/min/1.73m^2^)	94.1 ± 5.6	92.6 ± 6.3	1.5	0.209
L1-L4 T-score	−2.92 ± 1.00	−1.97 ± 0.95	−0.05	0.699
Femur neck T-score	−1.93 ± 0.65	−1.87 ± 0.77	−0.05	0.655
Femur hip T-score	−1.46 ± 0.66	−1.43 ± 0.58	−0.03	0.774

*Basal, patients evaluated at the enrolment; TZD, patients evaluated on TZD treatment; Δ Basal-treated, the difference between basal and treated values; n, number; Ca, total calcium; P, phosphate; PTH, parathormone; 25OHD, 25hydroxyvitamin D; UCa, urine calcium excretion; eGFR, estimated glomerular filtration rate calculated according to the KDIGO CKD-EPI formula (17). Normally distributed variables are presented as mean ± SD and analyzed by T-test of Student's test; non-normally distributed variables are presented as median (range interquartile) and analyzed by Mann–Whitney test; P-value with statistical significance is indicated in bold*.

#### Effects of TZD on Mineral Metabolic Profile

We analyzed the effect of the TZD treatment in the three mineral metabolic profiles identified by cluster analysis at basal evaluation (Table 5). TZD treatment induced an efficient reduction of the UCa independently from the cluster in which patients were included. The TZD-induced reduction in UCa was associated with the reduced PTH levels in patients included in clusters 1 and 2A, while any change was observed in patients included in cluster 2B, who had circulating PTH levels in the normal range. TZD treatment did not induce any significant change in serum calcium and phosphate levels in patients of the three clusters.

**Table 5:** Effects of thiazidic diuretics (TZD) treatment on clinical and metabolic parameters in 49 postmenopausal women treated with TZD.

	**CLUSTER 1**			**CLUSTER 2**					
				***CLUSTER 2A***			***CLUSTER 2B***		
**Parameters**	**Basal**	**TZD**	**P**	**Basal**	**TZD**	**P**	**Basal**	**TZD**	**P**
*n*	7	7		13	13		29	29	
Follow up (months)		48.0 ± 33.5			25.1 ± 15.1*			31.8 ± 15.3	
Age (years)	63.1 ± 6.9			61.7 ± 6.6			61.6 ± 7.0		
BMI (kg/m^2^)	28.3 ± 5.0			26.7 ± 5.3			24.4 ± 4.5		0.107
Cholecalciferol (%)	5 (71)	6 (86)	0.633	9 (69)	13 (100)	0.096	20 (69)	29 (100)	**0.002**
Bisphosphonates (%)	2 (29)	0 (0)	1.000	5 (38)	4 (31)	1.000	6 (21)	9 (31)	0.550
Denosumab (%)	0 (0)	3 (43)	0.192	0 (0)	4 (31)	0.096	1 (5)	7 (24)	0.052
Serum Ca (mg/dl)	9.8 ± 0.7	10.1 (9.3, 10.1)	0.597	9.6 ± 0.7	9.7 (9.5, 10.1)	0.105	9.7 ± 0.5	9.5 (9.1, 9.9)	0.358
Serum P (mg/dl)	2.8 (2.5, 3.8)	2.7 ± 0.7	0.468	3.4 (2.7, 3.8)	3.2 ± 0.6	0.387	3.4 (3.1, 3.7)	3.4 ± 0.4 §	0.266
Plasma PTH (pg/ml)	145.6 ± 38.9**	106.9 ± 63.3	**0.017**	83.3 ± 11.2**	64.7 ± 23.4°	**0.009**	47.7 ± 13.3**	47.2 ± 16.8°	0.745
Serum 25OHD (ng/ml)	39.9 ± 14.0	46.0 (28.8, 63.2)	0.394	31.1 ± 9.6	41.8 (30.1, 51.1)	**0.036**	37.7 ± 14.6	41.5 (35.1, 48.4)	**0.018**
eGFR (ml/min/1.73m^2^)	94.7 (93.1, 95.7)			94.7 (90.5, 98.8)			93.6 (88.9, 99.0)		
UCa (mg/24h)	422.2 (384.0, 504.0)	253.5 ± 100.3	**0.003**	372.0 (312.2, 454.5)	263.0 ± 83.8	**<0.001**	313.7 (270.5, 371.0)#	204.7 ± 78.3	**<0.001**
UCa (mg/kg/24 h)	6.42 ± 1.94	3.72 ± 1.82	**0.005**	5.79 ± 1.01	4.01 ± 1.11	**<0.001**	5.74 ± 1.58	3.40 ± 1.27	**<0.001**
Kidney stones (%)	4 (57.1)			3 (23.0)			13 (44.8)		

*n, number; BMI, body mass index (kg/m^2^), Ca, total calcium; P, phosphate; PTH, parathormone; 25OHD, 25hydroxyvitamin D; eGFR, estimated glomerular filtration rate calculated according the KDIGO CKD-EPI formula (17); UCa, urine calcium excretion. Normally distributed variables are presented as mean±standard deviation and analyzed by T-test of Student; non normally distributed variables are presented as median (range interquartile) and analyzed by Mann-Whitney test; P-value with statistical significance are indicated in bold*.

* *P = 0.035 vs. Cluster 1 at basal evaluation by ordinary One-way ANOVA*.

** *P < 0.05 among Cluster 1, Cluster A and Cluster B by ordinary One-way ANOVA*.

§ *P = 0.013 vs. Cluster 1 at on treatment evaluation by ordinary One-way ANOVA*.

° *P < 0.01 vs. Cluster 1 at on treatment evaluation by ordinary One-way ANOVA*.

# *P = 0.016 vs. Cluster 1 at basal evaluation by Dunn's multiple comparison test*.

#### Effects of TZD on Bone Mineral Density

Bone mineral densities at lumbar and femur sites did not vary significantly after TZD treatment in patients without concomitant anti-resorptive drugs (Table 4).

Of note, considering the subset of women concomitantly treated with TZD and anti-resorptives (bisphosphonates or denosumab, *n* = 25), we found that patients gaining normalization of the UCa (UCa <4 mg/kg/24 h; *n* = 17) experienced significant increases in bone mineral densities at lumbar (L1–L4 T-score −3.12 ± 1.11 vs. −2.85 ± 1.09, *P* = 0.032) and femur neck (femur neck T-score −2.43 ± 0.62 vs. −2.23 ± 0.75, *P* = 0.077). By contrast, patients with persistent hypercalciuria on TZD treatment did not show significant changes in bone mineral densities (L1–L4 T-score −2.99 ± 1.04 vs. −2.95 ± 1.24, *P* = 0.903; femur neck T-score −2.31 ± 0.77 vs. −2.61 ± 0.70, *P* = 0.319).

## Discussion

We retrospectively investigated a consistent series of postmenopausal women with osteopenia/osteoporosis and persistent hypercalciuria in the presence of sufficient 25OHD levels (19). Kidney stones occurred in one-third of patients, whose UCa did not differ from that in women who did not experience kidney stones, suggesting that the screening for hypercalciuria should be performed also in patients with no history of recurrent kidney stones. Lithogenesis is associated with individual and environmental factors. Individual factors include metabolic disturbance, inheritance, urinary tract infection, and obstruction (20). Of note, in patients with primary hyperparathyroidism, relative hyperoxaluria (21), hypomagnesuria (22) as well as polymorphic variants of the gene encoding the calcium sensing receptor (23) have been identified as factors significantly associated with kidney stones.

Unsupervised hierarchical clustering analysis identified a spectrum of mineral metabolic profiles in hypercalciuric women with reduced bone mineral density, similar to what was reported in patients with nephrolithiasis (10). Mineral metabolic profiles range from features resembling PHPT with reduced phosphate levels (cluster 1) to hyperparathyroidism with normal phosphate levels (cluster 2A) to profile with all mineral parameters within the normal range (cluster 2B). PTH was the main determinant of the mineral metabolic profile associated with hypercalciuria and reduced bone mineral density. The detection of low phosphate levels in the presence of hyperparathyroidism is consistent with the phosphaturic action of PTH at kidney level; the concomitant tendency to higher calcium levels and higher renal calcium excretion were suggestive of a certain degree of inappropriate PTH secretion, identifying a condition distinct from that detected in cluster 2A patients, were increased PTH levels were likely secondary to hypercalciuria. Indeed, it should be noted that the mineral metabolic profile in patients included in clusters 1 and 2A showed considerable overlap. Of note, in the present series of hypercalciuric postmenopausal osteoporotic women, hypercalcemic PHPT is rare, suggesting that diagnosis of normocalcemic or hypercalcemic PHPT needs to be carefully evaluated as well as an indication to parathyroid surgery. Unfortunately, any biochemical or clinical parameters correlated with the mineral metabolic profile.

We further investigated the effect of TZD treatment in this series of patients. TZD effectively reduced renal calcium excretion with a mean decrease of 39% of basal levels and determined a normalization of UCa in about two-thirds of patients. This pattern of response to TZD treatment was confirmed in the subgroup of patients free from anti-resorptive drugs. Of note, in the whole series of TZD treated patients, concomitant treatment with denosumab contribute to further reduction of UCa, while bisphosphonates were neutral. Moreover, TZD treatment reduces the PTH levels in patients with hyperparathyroidism, suggesting that the increased PTH levels are at least in part secondary to hypercalciuria. This is in line with what was reported by Eisner et al. (24), who proposed the “thiazide challenge” test to discriminate between primary and secondary hyperparathyroidism in patients with recurrent kidney stones based on normalization of PTH levels after administration of hydrochlorotiazide 25 mg two times a day for 2 weeks. Indeed, patients in the present series were treated with lower doses of TZD. Considering the whole series, we observed that anti-resorptive drugs may blunt the TZD-induced reduction of the PTH levels.

The follow-up of TZD treated patients, though of variable duration in every single patient, showed that bone mineral densities at both lumbar and femur sites were unaffected by TZD treatment alone. Of interest, patients with TZD-induced normalization of UCa experienced a significant increase of bone mineral densities when concomitantly treated with anti-resorptive drugs, while such positive effect could not be detected in patients with persistent hypercalciuria. Though coming from a series of limited sample size, data support the utility to treat hypercalciuria with TZD in hypercalciuric postmenopausal women with osteopenia/osteoporosis in addition to anti-resorptives when the risk of fracture is increased. Of note, present data suggest that denosumab may contribute to reducing UCa, while bisphosphonates have no effect.

Admittedly, the retrospective design of the study represents a limitation as well as the heterogeneity of the hydrochlorothiazide and indapamide doses and the anti-resorptive treatment. Moreover, the real-life setting prevented properly assessing the dietary calcium intake. Nonetheless, we provide data from the real-life setting about clinical and biochemical features of hypercalciuria and the efficacy of treatment with TZD and anti-resorptives, which highlighted the clinical role of hypercalciuria in the management of patients with osteoporosis and prompted the need for randomized controlled studies.

In conclusion, in hypercalciuric postmenopausal women with reduced bone mineral density, alterations of the mineral metabolic profile may represent a diagnostic challenge in distinguishing PHPT from secondary hyperparathyroidism. In a real-life clinical setting treatment with TZD is effective in reducing and normalizing UCa and PTH levels with any significant difference between indapamide and hydrochlorotiazide. Of note, patients with normalized urine calcium excretion by TZD treatment experienced significant increases in bone mineral densities when treated with anti-resorptives, bisphosphonates, or denosumab.

## Data Availability Statement

The datasets presented in this study can be found in online repositories. The names of the repository/repositories and accession number(s) can be found at: https://doi.org/10.5281/zenodo.5516995.

## Ethics Statement

The studies involving human participants were reviewed and approved by IRCCS Ospedale San Raffaele Milano. The patients/participants provided their written informed consent to participate in this study.

## Author Contributions

FN collected and analyzed data. GD, GG, and ML managed patients. SC conceived the study, reviewed the statistical analysis, and wrote the manuscript. All authors reviewed, discussed, and approved the final version of the manuscript.

## Funding

The study was supported by the GSD Foundation 5x1000 fund to Osteoregistry and the Italian Ministry of Health.

## Conflict of Interest

The authors declare that the research was conducted in the absence of any commercial or financial relationships that could be construed as a potential conflict of interest.

## Publisher's Note

All claims expressed in this article are solely those of the authors and do not necessarily represent those of their affiliated organizations, or those of the publisher, the editors and the reviewers. Any product that may be evaluated in this article, or claim that may be made by its manufacturer, is not guaranteed or endorsed by the publisher.
